# Oxytropis falcata bunge extract combined with black soybean oil ameliorates DNCB-induced atopic dermatitis-like skin inflammation

**DOI:** 10.3389/fphar.2025.1549492

**Published:** 2025-06-19

**Authors:** Rupei Chen, Yan Wu, Xianwei Wu, Jianhua Bu, Meng Liu, Qianya Zhao, Yan Chen, Jitao Tian, Jinjin Kai

**Affiliations:** ^1^ The First School of Clinical Medicine, Gansu University of Chinese Medicine, Lanzhou, China; ^2^ Department of Dermatology, Gansu Provincial Hospital, Lanzhou, China

**Keywords:** Oxytropis falcata bunge, black soybean, atopic dermatitis, HDAC3, NF-κB

## Abstract

**Background:**

*Oxytropis falcata* Bunge (OF) is a traditional Tibetan medicine, while black soybean oil (BSO) is a contemporary treatment used for eczema. Pharmacological studies have indicated that both exhibit strong anti-inflammatory effects. However, the role of OF in atopic dermatitis remains uncertain.

**Objective:**

To investigate the anti-inflammatory effects and underlying mechanisms of *O. falcata* Bunge extracts (OFE) and BSO in DNCB-induced atopic dermatitis in mice.

**Methods:**

Mice were divided into six groups: positive control (1% mometasone furoate), OFE, BSO, OFE + BSO, DNCB, and control. After 20 days of local application of each ointment, therapeutic effects were evaluated. Histopathological examination was performed to assess skin thickness and mast cell infiltration. ELISA was used to quantify proinflammatory cytokines, real-time PCR measured IL-36 mRNA levels, and Western blotting analyzed HDAC3/NF-κB and CysLTR1 protein expression.

**Results:**

All treatments alleviated DNCB-induced atopic dermatitis symptoms, with the combination group showing the most significant improvement in epidermal thickness, mast cell infiltration, and dermatitis severity. In addition, the treatment groups suppressed activation of the HDAC3/NF-κB signaling pathway.

**Conclusion:**

The combination of OFE and BSO can effectively reduce DNCB-induced atopic dermatitis in mice. Its action does not rely on broad immunosuppression or induce skin toxicity and may involve inhibition of proinflammatory cytokine release and downregulation of the HDAC3/NF-κB signaling pathway.

## 1 Introduction

Atopic dermatitis (AD) is a chronic, relapsing inflammatory condition and the most prevalent form of eczema, often referred to as atopic eczema (Chovatiya, 2023). Over the past decades, its global incidence has shown a significant increase, with a prevalence ranging between 10% and 21% worldwide (Paller et al., 2019), affecting up to 20% of children and 10% of adults (Williams and Flohr, 2006). Patients with AD frequently experience recurrent dry, scaly, and erythematous lesions accompanied by severe pruritus, which imposes considerable physical and psychological burden. Currently, topical glucocorticosteroids remain the first-line clinical treatment. However, their use is limited due to significant side effects and unsuitability for long-term therapy. Systemic treatments are also in use but carry similar concerns (Wollenberg and Ehmann, 2012).

AD is a multifactorial disorder. Its pathogenesis is closely associated with immune dysregulation, chronic inflammation, and impaired skin barrier function. Infiltration by CD4^+^T cells plays a central role, driving sustained inflammation and disrupting the balance between Th1 and Th2 responses (Langan et al., 2020). This immune imbalance initiates a cascade of inflammatory events. Epithelial cells release pro-inflammatory cytokines such as IL-4 and IL-13, which in turn stimulate IgE production by B cells. IL-36, secreted by keratinocytes, further promotes B cell class switching and IgE generation (Patrick et al., 2021). LTB4, a product of arachidonic acid metabolism, contributes to T-cell-driven inflammation by activating NF-κB signaling and increasing keratinocyte-derived CCL27 expression (Kanda and Watanabe, 2007). Mast cells also play a critical role in AD pathogenesis. They respond to IgE-mediated stimuli by releasing bioactive metabolites such as histamine, which accelerates disease progression (Numata et al., 2022).

Traditional Chinese Medicine (TCM) has long been recognized for its potential to treat diseases with a favorable safety profile (Wang, Z. et al., 2021). Tibetan medicine, a distinctive branch of TCM, is gradually gaining broader acceptance (Luo, 2015). *Oxytropis falcata* Bunge (OF) is a perennial, stemless herb and one of the three primary anti-inflammatory agents in Tibetan medicine. Traditionally used to clear heat, relieve toxicity, and reduce pain, it is mainly found in the northwestern region of Gansu. With a documented use spanning over 2,000 years, references to sickle-shaped *Oxytropis* appear in both the *Jingzhu Bencao* and *Chinese Materia Medica*, where it is referred to as the “King of Herbs” (Li et al., 2009). Today, a number of clinically applied formulations contain its active metabolites, such as Qingpeng Ointment, which is used to manage rheumatoid arthritis and pain, and Qizheng Xiaotong plaster (Zhang et al., 2020). OF, along with other species in the *Oxytropis* DC genus, remains widely utilized in Tibetan and traditional folk medicine. Flavonoids are considered its primary bioactive metabolites. Notably, three new flavonoids, echinacoside A, echinacoside B, and (6aR, 11aR)-3,8-dihydroxy-9,10-dimethoxypterocarpane, have been identified (Chen et al., 2010), and a total of 115 flavonoids have been isolated from both the aerial parts and roots (Guo et al., 2022), suggesting flavonoids are its dominant metabolites. OF is known to activate the hypothalamic-pituitary-adrenal axis, reduce the body’s stress response, and exert anti-inflammatory effects (Amirkhanova and Ustenova, 2018). Existing research has demonstrated its anti-inflammatory and analgesic actions (Zhang et al., 2020), along with antioxidant (Zhang et al., 2020), antitumor (Sheng et al., 2015), antimicrobial (Wang, B. et al., 2024), hypoglycemic (Yang et al., 2017), antifibrotic in idiopathic pulmonary fibrosis (Li et al., 2022; Wang, Y.-J. et al., 2021), and protective effects against myocardial ischemia-reperfusion injury (Guo et al., 2022; Zhang DeJun et al., 2017).

Black soybean (BS), a variant of soybean, is rich in bioactive substances. Metabolomic studies have revealed that black soybeans contain higher concentrations of anthocyanins compared to other colored varieties. They also possess high levels of polyphenols and exhibit the strongest antioxidant capacity among soybeans (Ganesan and Xu, 2017; Kumar et al., 2023). Prior studies have shown that extracts from black soybean possess anti-inflammatory, antioxidant (Kumar et al., 2023), hypoglycemic (Kurimoto et al., 2013), lipid-lowering, and antitumor (Chen et al., 2019) properties. Black soybean distillate oil (BSO), an oily metabolite derived from the dry distillation of black soybeans, has already been formulated into ointments for clinical application.

In this study, we observed that both OFE and BSO effectively reduced skin lesions and pathological alterations in mice with DNCB-induced AD-like symptoms, as reflected by decreases in swelling, erythema, dryness, and epidermal thickness. The combination of OFE with BSO produced a stronger effect, further suppressing splenic inflammatory infiltration in DNCB-treated mice. These effects were primarily linked to the capacity of OFE and BSO to reduce mast cell infiltration, inhibit the HDAC3/NF-κB signaling pathway, correct Th2 system imbalance, and influence immune function and lipid mediator activity, thereby reducing skin inflammation in this AD-like model. This study aimed to examine the potential mechanisms involved, particularly focusing on anti-inflammation and immunomodulation.

## 2 Materials and methods

### 2.1 Plant material and reagents

*Oxytropis falcata* Bunge [Fabaceae; *Oxytropis* DC] samples were collected from Maqu County, Gansu Province (Gansu, China), and the extract was identified by the College of Pharmacy, Gansu University of Chinese Medicine (Lanzhou, China). Black soybean distillate ointment was obtained from Xi’an Kanghua Pharmaceutical Co., Ltd. (Xi’an, China). 2,4-Dinitrochlorobenzene (DNCB) was purchased from Sigma-Aldrich (St. Louis, MO, United States). The solvent matrix (acetone:olive oil = 3:1) was purchased from Shanghai Macklin Biochemical Technology Co., Ltd. (Shanghai, China). Mometasone furoate (MF) cream was sourced from Shanghai Xinya Pharmaceutical Minhang Co., Ltd. (Shanghai, China), and white Vaseline cream was purchased from Henan Huakai Biotechnology Co., Ltd. (Henan, China).

### 2.2 Preparation of testing drugs and reagents

The extract of *O. falcata* Bunge was prepared using the ethanol extraction method. Dried plant material was ground and placed into a round-bottom flask, followed by the addition of ethanol. The mixture was heated and refluxed for 3 h. The extract was filtered, concentrated under reduced pressure, and freeze-dried to obtain the total flavonoids. Flavonoids were known to be the most abundant metabolites in the Oxytropis falcata Bunge extract (Yang et al., 2017). Furthermore, our previous study determined that 2′,4′-dihydroxychalcone constituted 6.25% of the total flavonoids extract from O. falcata. Consequently, flavonoids primarily account for the potent anti-inflammatory and antioxidant effects observed in the O. falcata extract. The resulting powder was blended with petroleum jelly under heated water bath conditions at 45°C to prepare a 5% (w/w) *O. falcata* Bunge extract ointment. For the BSO formulation, a 10% black soybean distillate oil ointment was diluted with petroleum jelly to make a 5% (w/w) BSO ointment. DNCB powder was dissolved in a 3:1 acetone-olive oil solution to prepare 2% and 0.5% (w/v) DNCB solutions, which were stored in airtight containers protected from light.

### 2.3 Animal experiments

#### 2.3.1 Animals

SPF-grade BALB/c mice (6–8 weeks old, 20 ± 2 g, with equal numbers of males and females) were purchased from Cavens Laboratory Animal Co., Ltd. (Changzhou, China). The mice were housed in the SPF experimental animal center at Gansu University of Chinese Medicine under standard laboratory conditions for a 4-day acclimation period. Environmental parameters were maintained at a 12-h light/dark cycle, 25°C ± 2°C temperature, and 40%–60% humidity, with food and water available *ad libitum*. All animal procedures were approved by the Ethics Committee of Gansu University of Chinese Medicine (SY2024-268).

#### 2.3.2 Acute toxicological tests

Skin irritation caused by the test formulations was assessed based on established methods (Fan et al., 2024). Four groups (n = 3) were designated for skin irritation evaluation. A 3 cm × 4 cm area on the dorsal skin of each mouse was shaved to expose the application site. Each group received daily topical application of one of the following for 14 consecutive days: Vaseline ointment (300 mg/d), 5% *O. falcata* Bunge extract ointment, or 5% *O. falcata* Bunge extract ointment combined with black soybean distillate ointment. Throughout the experimental period, mice were monitored for signs of skin abnormalities, behavioral changes, exophthalmos, and mortality. Body weights were recorded every 7 days. Two hours after the final administration, all animals were euthanized, and skin samples were collected for pathological examination using HE staining.

#### 2.3.3 Establishment of an atopic dermatitis-like mouse model and OFE/BSO intervention

**FIGURE 1:**
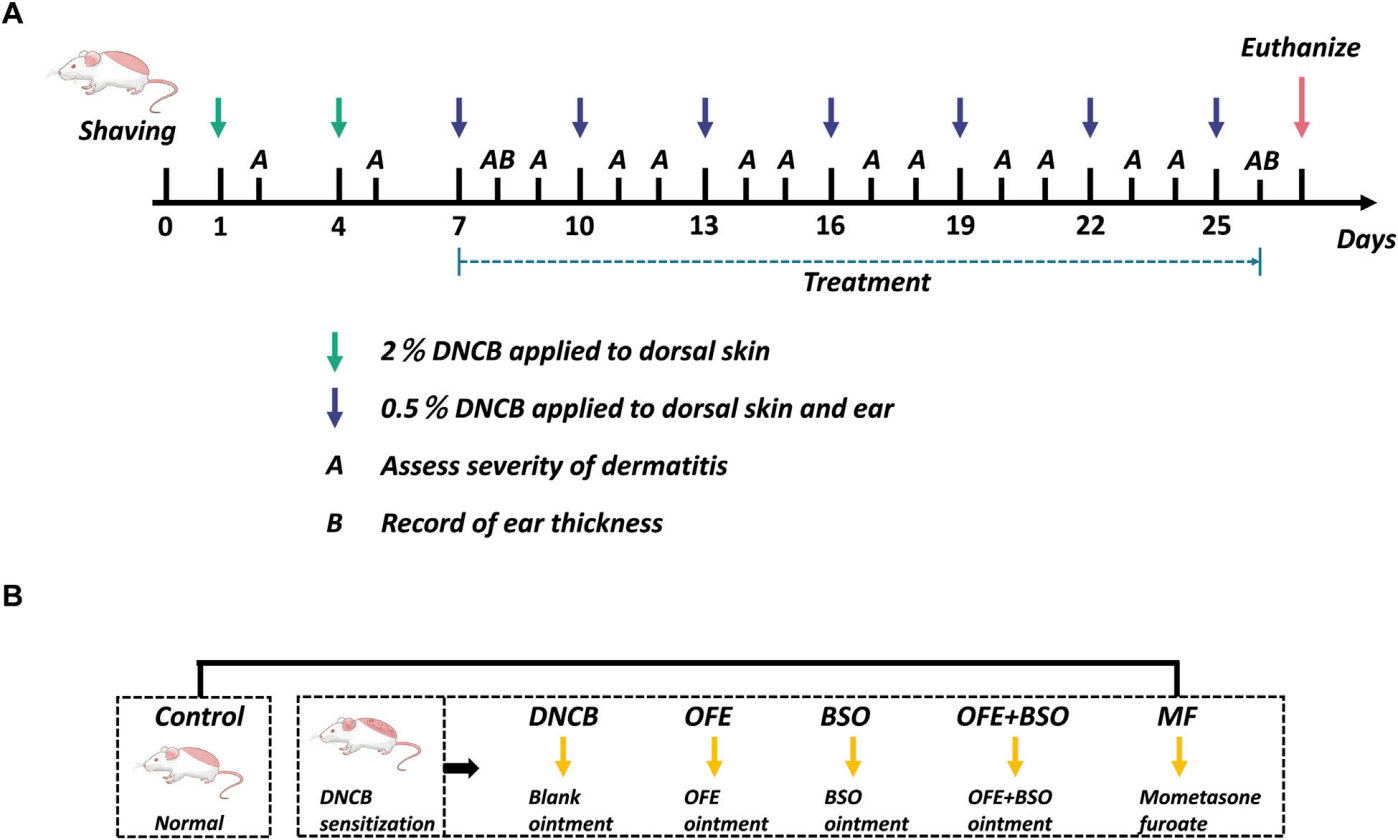
Overview of the grouping and experimental procedures in this study. **(A)** On days 1 and 4, 2% DNCB was applied to the shaved area to sensitize AD skin inflammation. Starting on day 7, 0.5% DNCB was given every 3 days to re-stimulate. Left ear thickness was recorded 2 h before administration on days 8 and 26, and dermatitis severity was assessed after each excitation. **(B)** BALB/c mice were divided into 6 groups: control, DNCB, OFE (300 mg/d), BSO (300 mg/d), OFE + BSO (300 mg/d), and MF.

As illustrated in Figure 1, 48 mice were randomly assigned to six groups (n = 8): Control group (Control), model group (DNCB), *O. falcata* Bunge extract group (OFE), black soybean distillate oil group (BSO), *O. falcata* Bunge extract + black soybean distillate oil group (OFE + BSO), and positive control group (MF). On Day 0, the dorsal hair was shaved and removed using depilatory cream to create a 3 cm × 4 cm exposed area. All mice, except those in the Control group, were sensitized with 120 μL of 2% (w/v) DNCB solution on days 1 and 4. From day 7, the dorsal skin and ears were challenged with 120 μL of 2% (w/v) DNCB and 20 μL of 0.5% (w/v) DNCB, respectively. This stimulation was repeated every 3 days for a total of seven applications (Han et al., 2022). Successful model establishment was confirmed by visible erythema, papules, and epidermal exfoliation. Beginning on day 7, 2 hours after DNCB application, each treatment group received daily topical administration of 300 mg/d of their designated ointment: 5% (w/w) OFE, 5% (w/w) BSO, or 5% OFE + BSO. The positive control group was treated with MF, and the Control group received the blank matrix. Skin lesions were scored on days 2 and 5, and following each subsequent DNCB challenge, using a scale based on erythema, papules/edema, exfoliation, and lichenification (0 = none, 1 = mild, 2 = moderate, 3 = severe). Body weight was recorded daily. Ear thickness was measured on day 8 and again before the final intervention. On day 27, mice were anesthetized, and blood samples were collected via orbital bleeding. After centrifugation, serum was separated and stored at −80°C. Skin samples (0.5 × 1.0 cm) from representative lesions were excised, with subcutaneous fat removed and tissue fixed in 4% paraformaldehyde. Remaining skin tissue was snap-frozen in liquid nitrogen and stored at −80°C in cryotubes. Spleens were isolated, cleared of surrounding tissue, weighed, and used to calculate the spleen index (spleen weight/body weight).

### 2.4 Histopathological analysis

Dorsal skin tissue samples from mice were fixed in 4% paraformaldehyde for 24 h. The tissues were then dehydrated, embedded in paraffin, and sectioned into 5 μm slices. Hematoxylin and eosin (H&E) staining was used to assess histopathological features, while toluidine blue staining was performed to evaluate mast cell infiltration. Microscopic images were captured using a Sunny Optical RX50 microscope. A Pannoramic MIDI II slide scanner was used for digital imaging, and ImageJ software was applied for quantitative analysis.

### 2.5 Enzyme-linked immunosorbent assay

Blood samples were collected via orbital puncture and allowed to clot for 1 h at room temperature. The serum was separated by centrifugation at 3,000 r/min for 15 min and stored at −80 °C. ELISA kits for IL-4, IL-36, IgE, LTB4, and CCL27 were purchased from Beijing Zhichao Weiye Biotech Co., Ltd. (Beijing, China). All procedures were carried out following the manufacturer’s protocols. OD values were measured at 450 nm, and standard curves were plotted using linear regression.

### 2.6 Real-time PCR analysis

After homogenizing skin lesions from the dorsal region, total RNA was extracted using the TRIzol method. cDNA was synthesized using the Evo M-MLV reverse transcription kit (Akerui, Hunan, China), following the manufacturer’s instructions. mRNA expression levels of target genes were measured using 2X SYBR Green Pro Taq HS Premix Ⅲ (Akerui, Hunan, China) under specified thermal cycling conditions. GAPDH served as the internal control. Relative gene expression levels were calculated using the 2^−ΔΔCT^ method. Primer sequences used in the analysis are provided in Table 1.

**TABLE 1:** Sequences of primers for Real-time PCR analysis.

Inflammatory gene	Forward primer	Reverse primer
IL-36	CTC​TTG​AGA​CGA​ACA​GGG​GG	ATG​TTC​CCT​TCC​CCA​AGC​TG

### 2.7 Western blotting analysis

Dorsal skin tissue was rapidly ground into powder using liquid nitrogen, followed by the addition of 0.5–1 mL pre-cooled RIPA lysis buffer (Cell Signaling Technology, MA, United States). The lysate was kept on ice for 10 min, mixed, and centrifuged to collect the supernatant containing total protein. Protein concentration was determined using a BCA assay kit (Cell Signaling Technology, MA, United States). Protein samples were denatured in SDS (Sigma-Aldrich, St. Louis, MO, United States), separated via electrophoresis on 10% polyacrylamide gels, and transferred onto PVDF membranes. The membranes were blocked with 5% nonfat milk at room temperature, then incubated overnight at 4 °C with primary antibodies: HDAC3, NF-κB (p65), and CYSLTR1 (Signalway Antibody, Maryland, United States), and GAPDH (Zhengneng, Chengdu, China) as loading control. After removing the primary antibody solution, membranes were washed three times with TBST (Tris-Borate-Sodium Tween-20), 5 min each wash. HRP-conjugated secondary antibodies were diluted with antibody diluent and applied. After incubation, membranes were washed again with TBST three times, 5 min each. Protein bands were visualized using ECL reagents (Millipore) and imaged using a chemiluminescent detection system.

### 2.8 Statistical analysis

Statistical analysis was conducted using GraphPad Prism 10.1.2. Data are presented as mean ± SEM. For comparisons among multiple groups, one-way ANOVA was used if the data followed a normal distribution. Comparisons between two groups were performed using Student’s t-test. Values of *p* < 0.05 were considered statistically significant.

## 3 Results

### 3.1 OFE, BSO, and OFE + BSO ointments produced no observable skin irritation in mice

**FIGURE 2:**
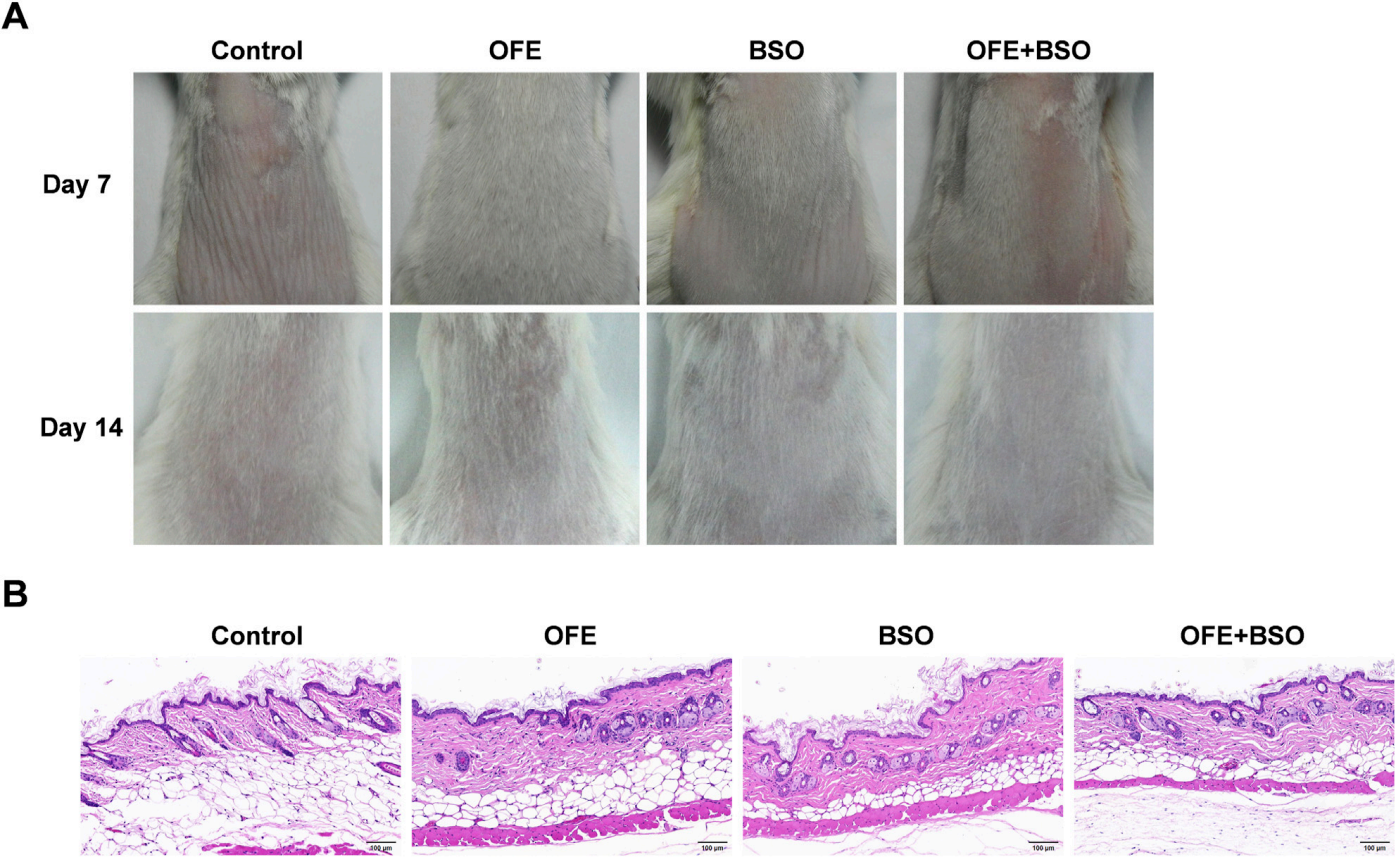
Results of skin irritation test. **(A)** Dorsal skin of mice in each group on the 7th and 14th day of administration. **(B)** HE staining of the dorsal skin of mice in each group after the last administration.

Topical skin irritation tests were performed using 5% OFE (300 mg/day), 5% BSO (300 mg/day), and 5% OFE + BSO (300 mg/day) ointments. No signs of skin lesions, weight loss, behavioral abnormalities, or mortality were observed in any group during the 14-day period (Figure 2A). All treatment groups exhibited faster hair regrowth compared to the control group, with the OFE group showing the most noticeable effect, although further confirmation is needed. Histopathological examination of the skin revealed no evident pathological changes following topical application of OFE, BSO, or OFE + BSO ointments (Figure 2B). Based on these findings, 5% (w/w) OFE, 5% (w/w) BSO, and 5% (w/w) OFE + BSO at 300 mg/day were selected as the intervention concentrations and doses for the subsequent efficacy and mechanism studies.

### 3.2 OFE, BSO, and OFE + BSO ameliorated the severity of dermatitis in AD mice

**FIGURE 3:**
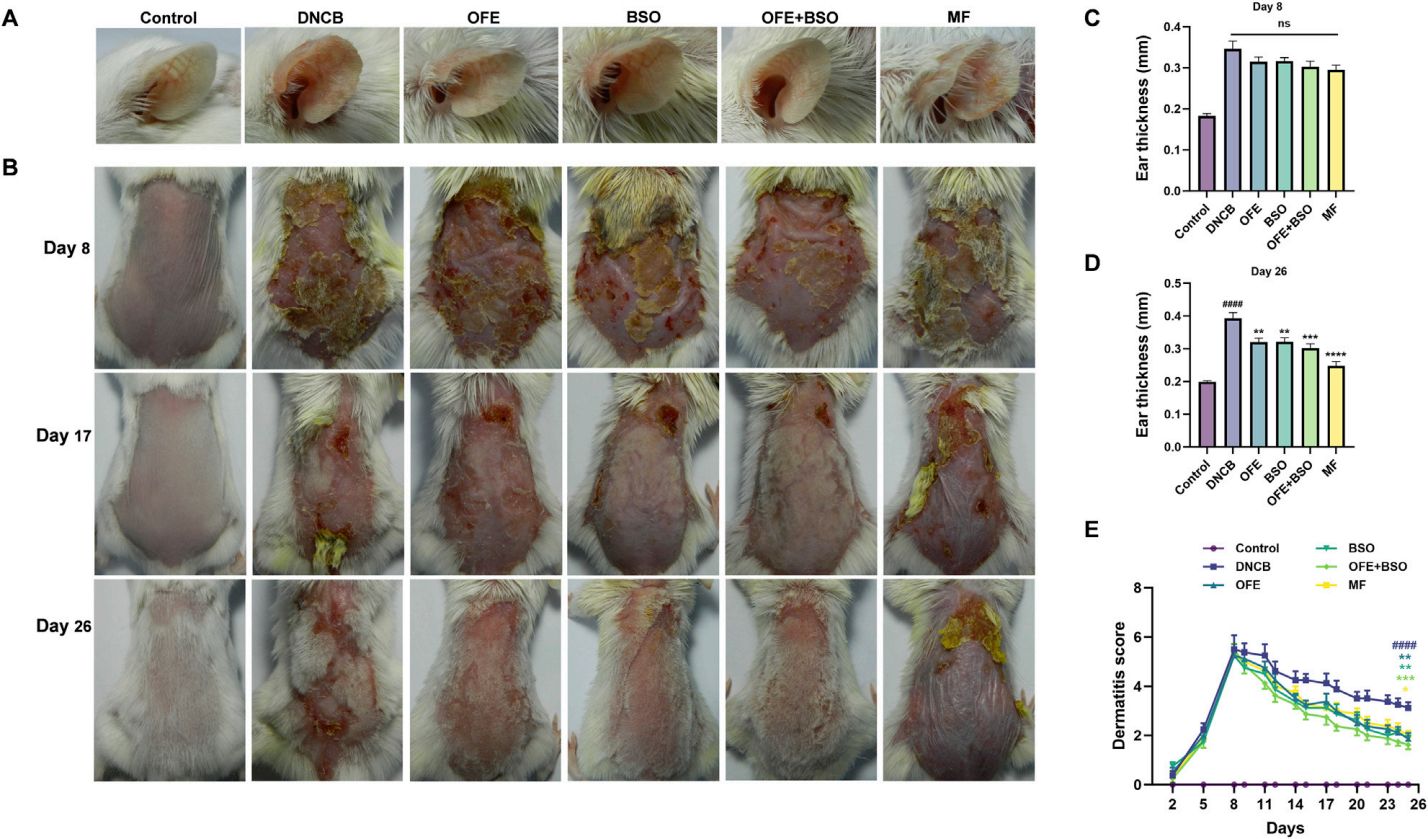
Changes in ear thickness and skin lesions in each group of mice. **(A)** Ear thickness of mice in each group on day 26. **(B)** Changes in skin lesions in each group of mice on days 8, 17 and 26 of the experiment. **(C)** Ear thickness on day 8 of the experiment. **(D)** Ear thickness on day 26. **(E)** Change in skin lesion score. n = 8, vs. control #p < 0.05; vs. DNCB group *p < 0.05.

**FIGURE 4:**
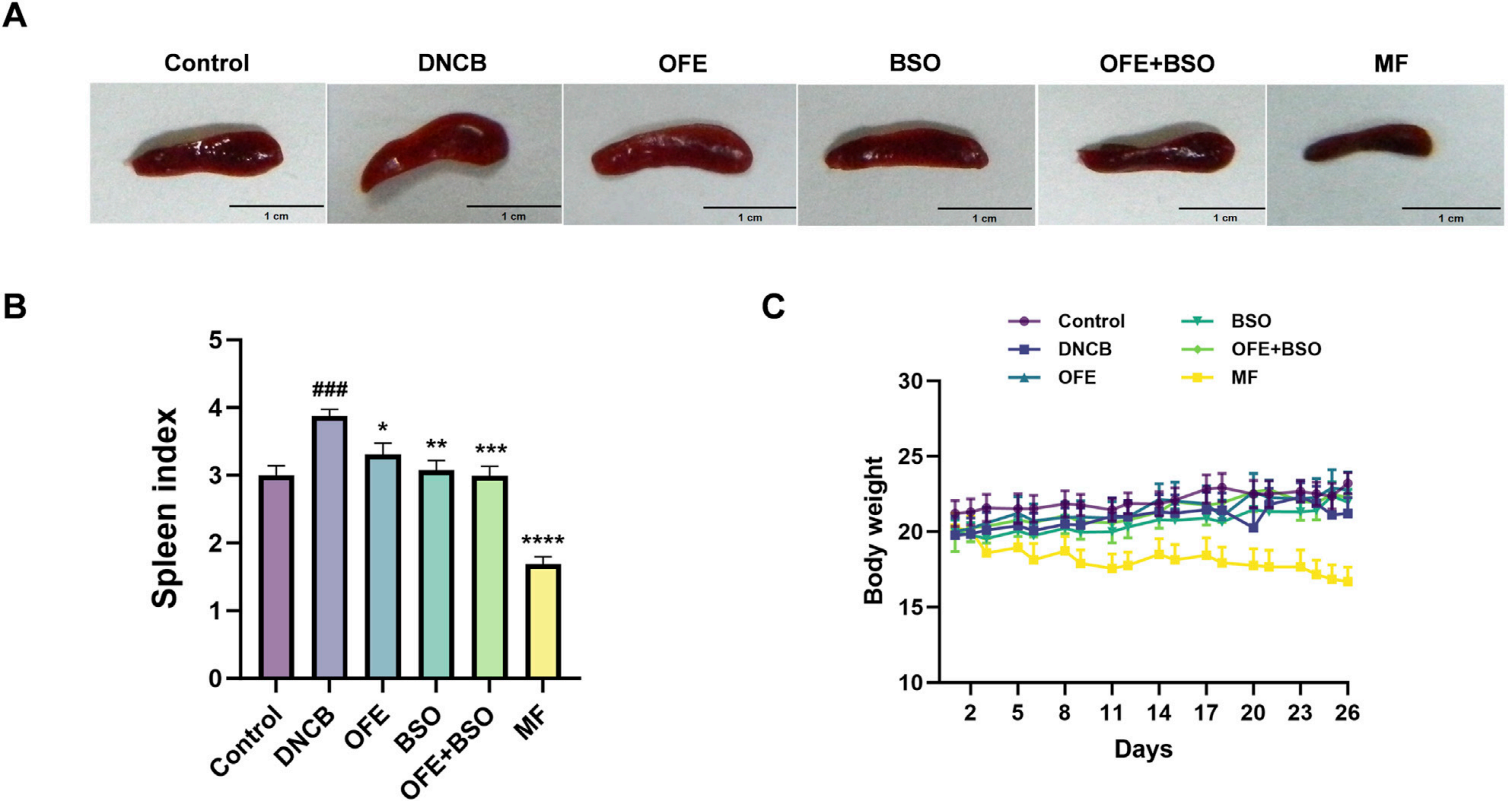
Changes in spleen and body weight of mice in each group. **(A)** Appearance of the spleen in mice 2 h after final administration. **(B)** Changes in splenic index of mice in each group. **(C)** Changes in body weight of mice in each group. n = 8, vs. control #p < 0.05; vs. DNCB group *p < 0.05.

A range of assessments was carried out to evaluate the effects of OFE, BSO, and OFE + BSO on atopic dermatitis-like symptoms in mice. The thickness of the left ear was measured at the beginning and end of the treatment period (Figures 3A,C,D). By day 26, all three treatment groups showed a significant reduction in ear thickness compared to measurements taken on day 8. Observations of the dorsal skin lesions revealed a clear reduction in the severity of dermatitis in the OFE, BSO, and OFE + BSO groups. Scoring of the skin lesions showed a significant drop across all three groups, with the OFE + BSO group showing the most significant improvement. This suggests that combining OFE with BSO may be more effective in reducing AD symptoms in mice (Figures 3B,E). Interestingly, the model group also showed a gradual decline in skin lesion scores over time, although the skin became increasingly thickened and developed a more lichenified appearance, indicating a shift toward chronic inflammation. Body weight was monitored throughout the treatment period. With the exception of the positive control group, mice in all other groups showed normal weight gain. The MF-treated group exhibited a significant decrease in body weight during the treatment phase. Spleen enlargement was observed in the model group, indicating immune activation. Treatment with OFE, BSO, and OFE + BSO partially reversed this enlargement. In contrast, MF treatment led to a significant reduction in spleen size and splenic index, suggesting immunosuppressive effects (Figure 4).

### 3.3 OFE, BSO, and OFE + BSO alleviate pathological changes in AD mice

**FIGURE 5:**
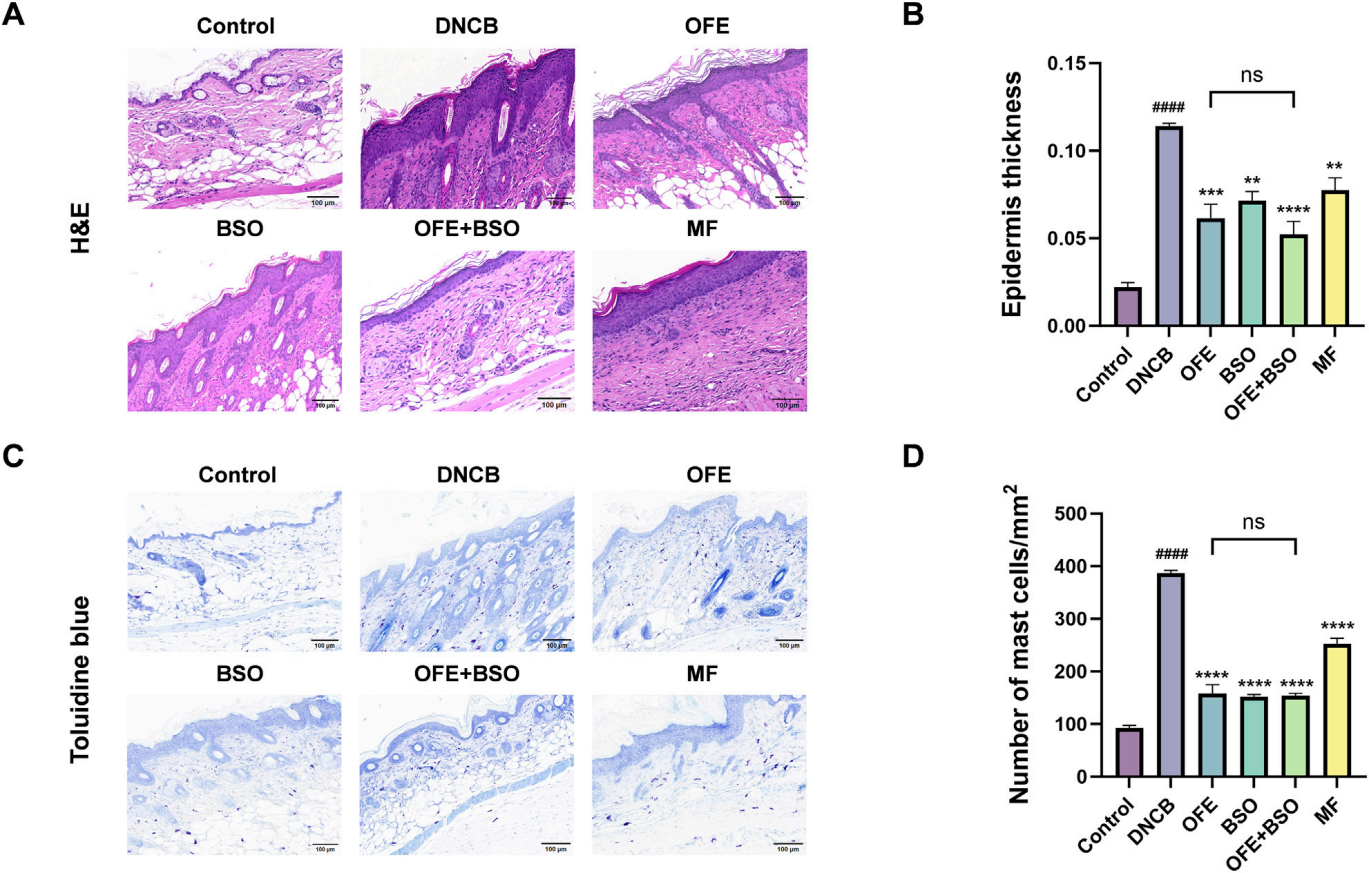
Histopathology of dorsal skin lesions in each group of mice. **(A)** HE staining of dorsal skin of mice in each group. **(B)** Degree of epidermal thickening in each group under HE staining. **(C)** Mouse back skin of each group after toluidine blue staining. **(D)** Number of mast cell infiltration in each group. n = 3, vs. control #p < 0.05; vs. DNCB group *p < 0.05.

Dorsal skin tissues from each group were stained with H&E and toluidine blue to assess histopathological changes. Compared with the control group, the model group exhibited clear pathological features, including stromal cell hyperplasia, epidermal thickening, hyperkeratosis, and infiltration of mixed inflammatory cells in the dermis and subcutaneous tissue. After 20 days of treatment, OFE, BSO, and OFE + BSO all significantly reduced inflammatory cell infiltration and epidermal thickening (Figures 5A,B). Notably, OFE showed a stronger effect on reducing epidermal thickness than BSO, while OFE + BSO showed the most pronounced improvement. Although no statistically significant difference in epidermal thickness was observed between the OFE+BSO combination group and individual monotherapies, a discernible trend toward reduced hyperplasia was noted. Toluidine blue staining revealed a significant increase in mast cell infiltration in the model group. Treatment with OFE, BSO, and especially OFE + BSO significantly reduced mast cell numbers (Figures 5C,D).

### 3.4 OFE, BSO, and OFE + BSO decreased inflammatory factors and IL-36 mRNA expression

**FIGURE 6:**
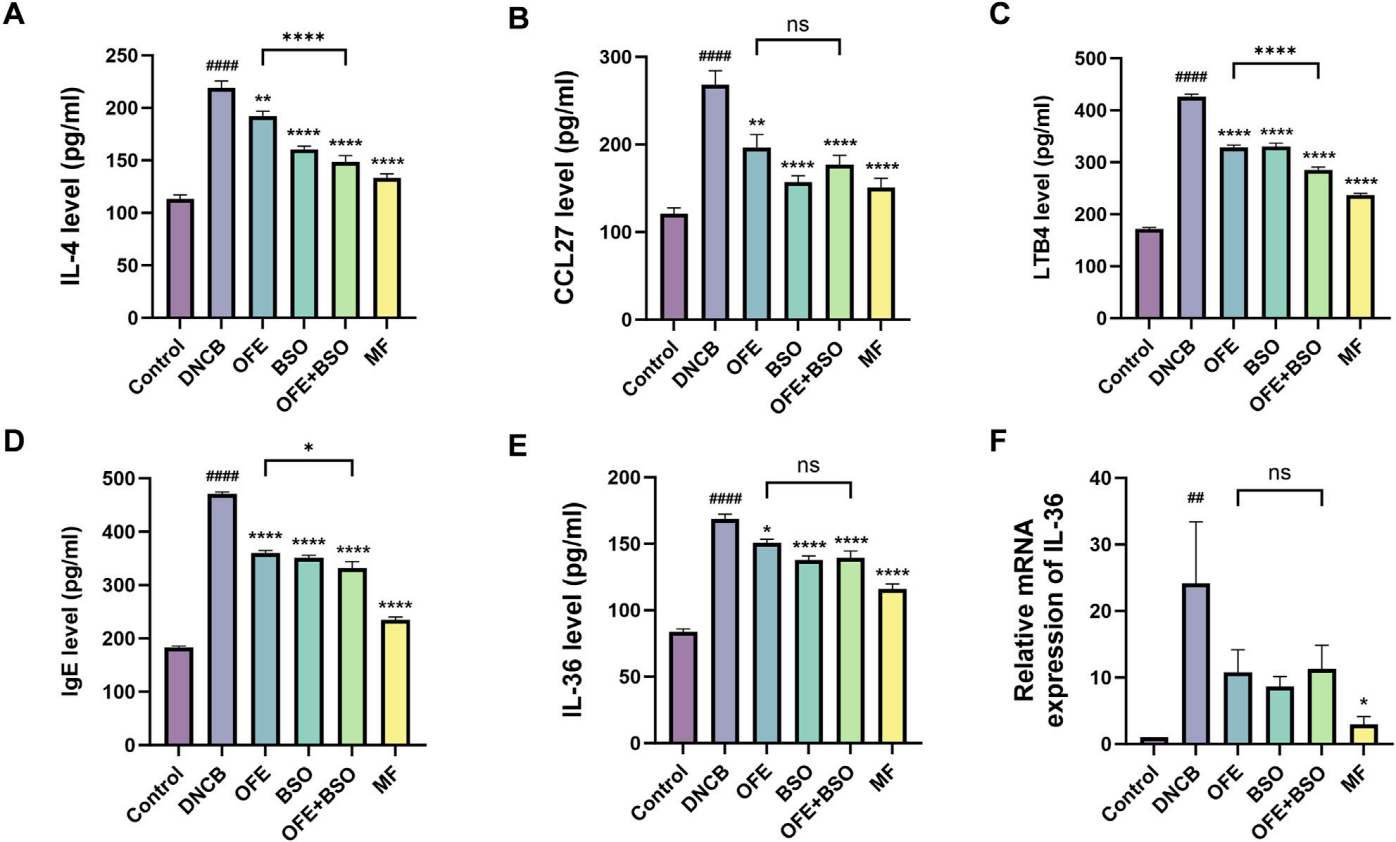
**(A -F)** Levels of inflammation-related factors in each group. Levels of IgE, lipid mediators (LTB4), Th2 cytokines (IL-4), pro-inflammatory cytokines (IL-36, CCL27) (n = 7) and IL-36 mRNA expression levels (n = 6) in each group. vs. control #p < 0.05; vs. DNCB group *p < 0.05.

Cytokine levels in serum and ear tissues were analyzed using ELISA and qPCR. The DNCB group displayed substantial cytokine dysregulation, including elevated levels of immunoglobulin E (IgE), lipid mediators (LTB4), Th2 cytokines (IL-4), and pro-inflammatory cytokines (IL-36, CCL27). Treatment with OFE, BSO, and OFE + BSO significantly reversed these elevations. The reduction in pro-inflammatory cytokines was more pronounced in the OFE + BSO group compared to the OFE group alone (Figure 6), suggesting that the combination may improve OFE absorption or effectiveness. Importantly, statistical analyses revealed significant differences in key inflammatory mediators between the OFE+BSO combination and OFE monotherapy groups, particularly for IL-4, LTB4, and IgE levels, findings that warrant prioritized investigation into their mechanistic basis and therapeutic implications. Although other pro-inflammatory factors did not reach statistical significance under current experimental conditions, notable trends toward reduction were observed. Furthermore, IL-36 mRNA expression was significantly upregulated in the DNCB group and significantly downregulated in all three treatment groups (OFE, BSO, and OFE + BSO) (Figure 6F).

### 3.5 OFE, BSO, and OFE + BSO inhibited the activation of HDAC3/NF-κB and the expression of CysLTR1

**FIGURE 7:**
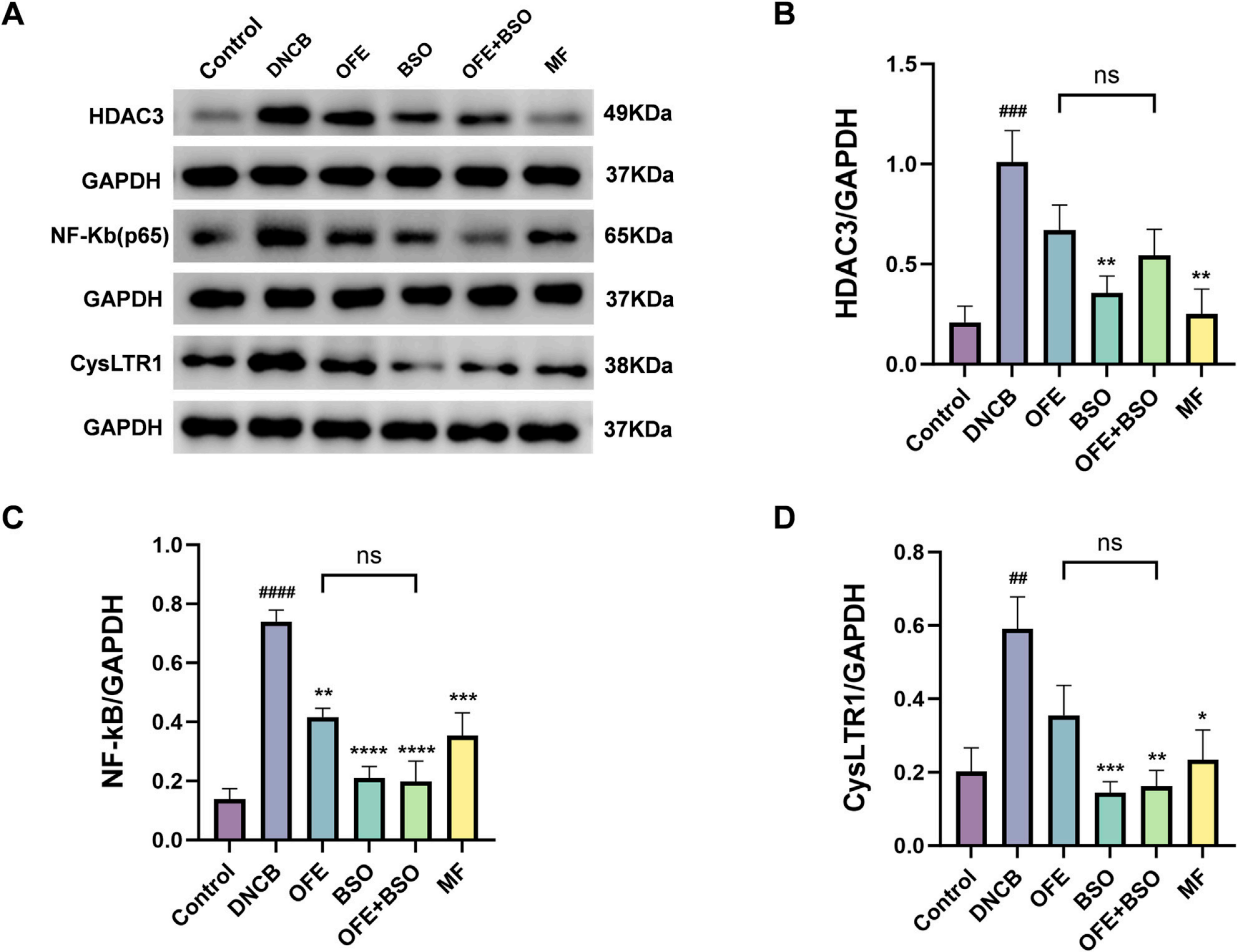
OFE, BSO, and OFE + BSO reduce protein expression of HDAC3/NF-κB and CysLTR1. **(A–D)** HDAC3/NF-κB and CysLTR1 protein levels in mice of each group. n = 6, vs. control #p < 0.05; vs. DNCB group *p < 0.05.

To investigate the potential signaling mechanisms involved in the effects of OFE, BSO, and OFE + BSO on atopic dermatitis, the expression levels of histone deacetylase 3 (HDAC3) and nuclear factor kappa B (NF-κB) in dorsal skin tissue were examined. In the DNCB group, HDAC3 and NF-κB activation was significantly increased. This activation was suppressed following treatment with OFE, BSO, and OFE + BSO. These findings suggest a strong association between HDAC3/NF-κB pathway activity and the inflammatory response observed in AD. The data indicate that OFE, BSO, and especially their combination may reduce atopic dermatitis symptoms by inhibiting HDAC3/NF-κB signaling. Additionally, the DNCB group showed significantly increased expression of CysLTR1 protein, which was also reduced by treatment with OFE, BSO, and OFE + BSO (Figure 7). While quantitative analysis of HDAC3/NF-κB pathway metabolites and CysLTR1 protein levels revealed no statistically significant differences between the OFE+BSO combination and OFE monotherapy groups, the combined treatment demonstrated a discernible trend toward inflammatory pathway modulation compared to OFE alone.

## 4 Discussion

*Oxytropis falcata* Bunge is a traditional Chinese medicinal herb known for its anti-inflammatory and antioxidant properties, commonly used for reducing fever and relieving pain. However, there is currently limited scientific evidence supporting its use in the treatment of AD. Black soybean distillate oil (BSO), which is formulated into ointments and frequently applied in the treatment of eczema, contains a high proportion of linoleic acid and possesses unique oily characteristics that may improve transdermal absorption (Zhong et al., 2015). Based on this, we explored the combined use of OFE and BSO in an AD mouse model to assess whether BSO could improve the skin permeability and availability of OFE. This study evaluated the effects of OFE, BSO, and their combination in a DNCB-induced AD-like mouse model. Treatment with these formulations led to visible improvement in skin inflammation. Histological findings further confirmed that all three treatments reduced epidermal thickening and mast cell infiltration, with the OFE + BSO group showing the most significant results. These outcomes suggest that OFE, BSO, and particularly their combination can reduce inflammatory cell infiltration and recruitment in the epidermis, contributing to an improved therapeutic effect in AD. Moreover, all three treatments were able to suppress activation of the HDAC3/NF-κB signaling pathway and reduce CysLTR1 expression. Serum levels of IgE, lipid mediator LTB4, Th2 cytokine IL-4, and pro-inflammatory cytokines IL-36 and CCL27 were also downregulated after treatment. These findings support the potential of OFE + BSO as a therapeutic strategy for atopic dermatitis. These findings preliminarily validate the synergistic therapeutic effects of the drug combination. However, confirmation of BSO-mediated enhancement in OFE transdermal permeability and bioavailability will require rigorous pharmacokinetic analyses, skin permeation studies, and comprehensive transdermal delivery data as suggested, which merits further investigation to fully substantiate these preliminary observations. This study still requires chronic toxicity evaluation, investigation of drug stability, and long-term efficacy tracking.

AD is a chronic, relapsing inflammatory disease that typically begins in infancy or childhood and tends to follow a prolonged, recurring course (Park et al., 2022). Its pathogenesis is primarily driven by inflammatory and type 2 immune responses, closely linked to disruptions in immune homeostasis. Under normal conditions, Th1 and Th2 cells maintain a dynamic balance. However, when the skin is exposed to AD-related triggers, this balance is disturbed, leading to an overactivation of Th2 responses. This imbalance initiates a cascade of inflammatory and immune events that contribute to disease progression. IL-4, a key cytokine secreted by Th2 cells, further promotes Th2 differentiation and drives B cell class switching, which increases IgE production. IL-4 also facilitates the recruitment of T cells and eosinophils into inflamed tissue, fueling the allergic response (Akdis et al., 2020). Mast cells, as frontline defenders of the immune system, play a vital role in detecting antigens and regulating physiological processes such as vasodilation. They help maintain *in vivo* immune homeostasis. Upon recognition of allergens by IgE bound to their surface, mast cells initiate signaling pathways that trigger the release of various cytokines and chemokines (Krystel-Whittemore et al., 2015). In this study, the DNCB-induced AD model showed extensive mast cell infiltration in skin tissue, along with elevated levels of IL-4 and IgE. These abnormalities were significantly reduced following treatment with OFE, BSO, and OFE + BSO treatments.

In the theory of paradoxical psoriasis, biologics targeting the Th2 system may induce an immune drift that drives Th17 transitions, with IL-36 playing a role in determining the clinical phenotype through quantitative differences in its expression (Maronese et al., 2024). In our study, we observed that IL-36 levels were elevated *in vivo* in DNCB-induced AD mice, and this increase was significantly reduced following treatment. This reduction suggests that the intervention may be more effective than biologics in controlling IL-36-driven inflammation. Keratinocytes are the primary cells forming the skin barrier and are now recognized not only as structural metabolites but also as active participants in AD pathogenesis. Studies have shown that keratinocytes contribute to the Th2-driven inflammatory process and can intensify disease severity (Gallegos-Alcalá et al., 2021). They produce a wide range of cytokines and chemokines. IL-36, a member of the IL-1 cytokine family, is secreted by keratinocytes and includes three agonists (IL-36α, IL-36β, IL-36γ) and one antagonist (IL-36Ra). IL-36α and IL-36γ are pro-inflammatory, while IL-36Ra has anti-inflammatory effects (Buhl and Wenzel, 2019). IL-36 is widely expressed at barrier sites in the body, particularly in epithelial and immune cells, where it plays roles in inflammation, tissue homeostasis, and barrier protection (Chi et al., 2017). It has been implicated in the transition of acute AD lesions to chronic states (Tsoi et al., 2020). Upon binding to the IL-36 receptor complex (IL-36R), agonists trigger NF-κB and MAPK activation, leading to T-cell proliferation and increased production of pro-inflammatory cytokines and chemokines (Müller et al., 2018). RNA sequencing analysis has confirmed higher IL-36 expression in lesional AD skin compared to non-lesional skin (He et al., 2021). In addition, keratinocytes produce CCL27 (CTACK), a chemokine also expressed in Langerhans cells (Morales et al., 1999). CCL27 plays a central role in T-cell–mediated inflammation by promoting T-cell homing. CCL27 levels are elevated in AD and correlate with disease severity (Homey et al., 2002; Holm et al., 2021). In our study, both IL-36 and CCL27 were upregulated in DNCB-induced AD mice.

Previous studies have reported elevated levels of LTB4 in patients with AD, suggesting its involvement in the inflammatory processes underlying the disease (Fogh et al., 1989; Ruzicka et al., 1986). LTB4 is a pro-inflammatory lipid mediator derived from arachidonic acid (AA) through enzymatic pathways involving 5-lipoxygenase (5-LOX) and cyclooxygenase (COX). These enzymes catalyze the oxidative metabolism of polyunsaturated fatty acids (PUFAs), generating lipid mediators that contribute to inflammation. Flavonoids from green tea have been shown to inhibit the enzymatic activity of both COX and LOX (Garg, 2020), highlighting a potential therapeutic angle for regulating lipid-mediated inflammation. Cysteinyl leukotriene receptors (CysLTRs), including CysLTR1 and CysLTR2, are expressed on the surface of various immune and inflammatory cells. Among them, CysLTR1 is more widely distributed *in vivo* and is expressed across multiple immune cell types (Kanaoka and Austen, 2019). CysLTR1 plays a role in the pathogenesis of asthma and allergic rhinitis (Theron et al., 2014), both of which often follow atopic dermatitis in the clinical progression known as the “atopic march” (Spergel and Paller, 2003). Targeting the CysLT signaling pathway has been proposed as a potential strategy to relieve inflammatory pruritus (Voisin et al., 2021). In the present study, LTB4 levels and CysLTR1 protein expression were significantly elevated in DNCB-induced AD mice. Treatment with OFE, BSO, and OFE + BSO ointments led to significant reductions in both, indicating a suppression of inflammatory signaling.

To further investigate the anti-inflammatory mechanisms of OFE, BSO, and OFE + BSO, we examined their effects on the HDAC3 and NF-κB signaling pathways. Histone deacetylase 3 (HDAC3) primarily regulates the deacetylation of acetylated histones and plays a role in epigenetic control. Previous studies have reported abnormal upregulation of HDAC3 in DNCB-induced lesional skin in AD mice, and selective inhibition of HDAC3 has been shown to relieve symptoms, suggesting its involvement in AD pathogenesis (Zhou et al., 2024), NF-κB is a central transcription factor that controls the expression of numerous inflammatory cytokines (Hayden et al., 2006). Its activation is triggered by upstream signaling complexes that lead to the activation of IKK. IKK phosphorylates IκBα, marking it for degradation, which frees the NF-κB p50/p65 heterodimer to translocate into the nucleus and initiate transcription of pro-inflammatory target genes (Lawrence, 2009). HDAC3 plays a critical role in this pathway by regulating the ability of NF-κB to bind to DNA promoters and activate transcription (Wang Y. et al., 2024). Previous work has shown that the anti-inflammatory metabolite butyrate can reduce HDAC3 expression and prevent the nuclear translocation of NF-κB p65 in AD mice (Hu et al., 2024). Additionally, blocking HDAC3 has been associated with reduced IL-33 expression and improved AD-like skin inflammation (Luo et al., 2023). In our study, both OFE and BSO significantly inhibited activation of the HDAC3/NF-κB pathway. The combination of OFE + BSO produced a stronger inhibitory effect than OFE alone. In this study, the alleviation of AD by OFE, BSO, and OFE+BSO through inhibition of the HDAC3/NF-κB pathway represents a preliminary investigation, necessitating further in-depth mechanistic exploration.

## 5 Conclusion

**FIGURE 8:**
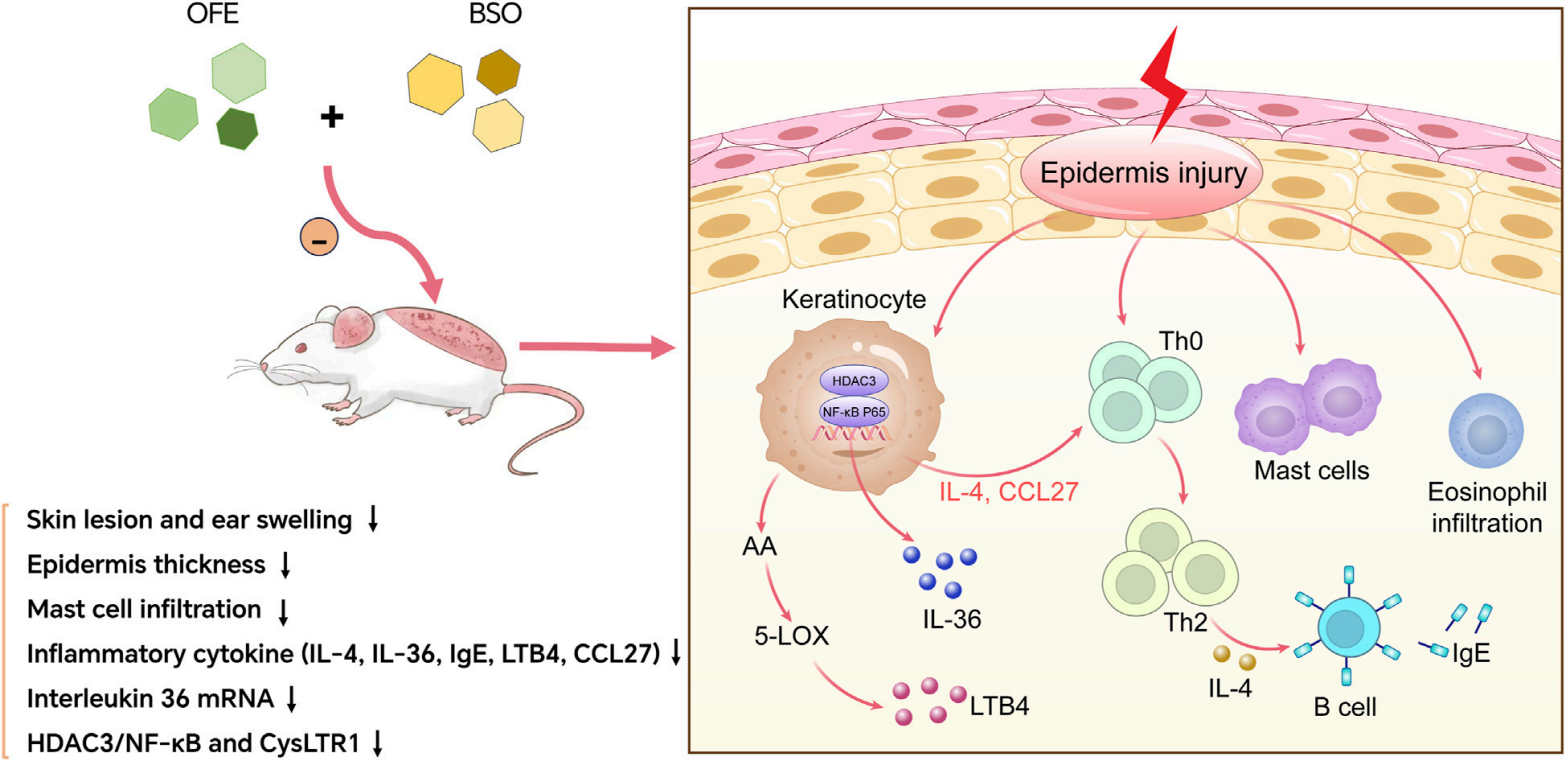
OFE, BSO, and OFE + BSO alleviate AD by inhibiting inflammatory factors and HDAC3/NF-κB signalling pathway.

Our findings showed that OFE, BSO, and their combination exerted distinct effects on DNCB-induced inflammatory responses in mice with AD. Both OFE and BSO reduced skin lesions, epidermal thickening, and mast cell infiltration. They also improved inflammatory responses by downregulating inflammation-related factors and suppressing activation of the HDAC3/NF-κB pathway. The combination of OFE and BSO appeared to increase the bioavailability of OFE, leading to a stronger anti-inflammatory effect. Importantly, no skin irritation was observed with any of the treatments. These results provide experimental support for the potential clinical application of the two drugs in the treatment of AD (Figure 8).

## Data Availability

The original contributions presented in the study are included in the article/Supplementary Material, further inquiries can be directed to the corresponding author.
